# Chemical Composition and In Vitro Antioxidant and Antimicrobial Activities of *Marrubium vulgare* L.

**DOI:** 10.1155/2021/7011493

**Published:** 2021-10-31

**Authors:** Ibrahim Mssillou, Abdelkrim Agour, Noureddine Hamamouch, Badiaa Lyoussi, Elhoussine Derwich

**Affiliations:** ^1^Laboratory of Natural Substances, Pharmacology, Environment, Modeling, Health and Quality of Life (SNAMOPEQ), Faculty of Sciences, Sidi Mohamed Ben Abdellah University, Fez 30 000, Morocco; ^2^Laboratory of Biotechnology and Plant Physiology, Faculty of Sciences, University Mohammed V, Rabat, Morocco; ^3^Unity of GC/MS and GC-FID, City of Innovation, Sidi Mohamed Ben Abdellah University, Fez 30 000, Morocco

## Abstract

In this study, the polyphenol content and the antioxidant and antimicrobial activities of hydroethanolic (MVE) and hydroacetonic (MVA) leaf extracts of *Marrubium vulgare* L. were examined. The results indicated that the total phenolic content was higher in MVA (112.09 ± 4.77 mg GAE/DW) compared to MVE extract (98.77 ± 1.68 mg GAE/DW). The total flavonoid content was also higher in MVA extract (21.08 ± 0.38 mg QE/g DW) compared to MVE (17.65 ± 0.73 mg QE/g DW). Analysis of the chemical composition revealed the presence of 13 compounds with a total of 96.14%, with the major compound being malic acid (22.57%). Both extracts possess a good total antioxidant activity. DPPH and FRAP assays indicated that the MVE extract possesses a better antioxidant activity, with IC_50_ = 52.04 *µ*g/mL ± 0.2 and EC_50_ of 4.51 ± 0.5 mg/mL, compared to MVA extract (IC_50_ = 60.57 ± 0.6 *µ*g/mL and EC_50_ of 6.43 ± 0.0411 mg/mL). Moreover, both extracts exhibited strong antimicrobial activity against certain nosocomial strains as indicted by the MIC values, which ranged between 0.93 mg/mL and 10 mg/mL. Taken together, these results reveal the importance of *M. vulgare* as a natural antioxidant with important antimicrobial activity.

## 1. Introduction

The problem of microbial resistance continues to increase in all regions of the world, and eventhough a very large number of antibiotics have been produced in the last 30 years, microbial resistance is maintained because of the wide use of these drugs against many infectious diseases [1]. The efficacy and use of herbal extracts as an antimicrobial agent are widely studied and reported in several studies [2]. Furthermore, medicinal plants are considered among the best natural source of antioxidants and widely studied and examined against several diseases linked to oxidative stress and high free radical rate, such as neurodegenerative disorders, cancer, and cardiovascular diseases [3].

Medicinal and aromatic plants (MAP) are known for their use in the treatment of different diseases and as food additives [4, 5]. They are rich in bioactive molecules with health benefits such as antioxidants and polyphenols [6, 7]. As pointed out by Edeoga et al. [8], phenolic compounds and tannins are the key phytochemical constituents in medicinal plants. Advantageous to both human and plant health, phenolic compounds including flavonoids should be prominent target in medicinal plant research. In plants, much of polyphenols importance traces to their roles in key functions such as antioxidant activity, free radical scavenging, signaling molecules, plant defense, and mediating auxin transport [9, 10].

The bioactive compounds of herbal plants have been well known to play an important role in the preservation of food and in protecting people against cardiovascular and chronic diseases [11, 12]. The Lamiaceae family contains essential oils and extracts with important pharmacological attributes and a rich content of bioactive constituents of biological interest [13, 14]. Among the most famous species of this family is an interesting plant called *M. vulgare* (white horehound) known in Morocco as “Merriwa,” a medicinal plant that is used in Morocco for its medicinal and curative effects. The plant is rich in chemical compounds such as polyphenols, saponins, and tannins and is widespread in the Mediterranean basin [15]. It has been reported that *M. vulgare* plant possesses antioxidant, antifungal, hypoglycemic, and hypotensive activities [16]. The plant also exhibits antibacterial, antispasmodic, antinociceptive, and insecticidal activities [17, 18]. *M. vulgare* also possess anticorrosion, anti-inflammatory, and vasorelaxant effects [19–21] and is considered as a good source of bioactive compounds with analgesic properties [22].

The current health problems linked with the use of substances of chemical origin, and the constraints of food preservation promoted us to look for alternatives in MAP. The objective of this study was to identify the chemical composition of *M. vulgare* leaves from Morocco and to evaluate the antioxidant and antimicrobial potential effects of its extracts against nosocomial pathogens.

## 2. Materials and Methods

### 2.1. Plant Material

*M. vulgare* was collected in the region of Fez 34°03′57.8″N 5°03′47.2″W in September 2020 and was identify by Pr. Bari Amina, a plant taxonomist at the Faculty of Sciences, University Sidi Mohamed Ben Abdellah, Fez. A voucher sample of the plant (RH001190122) was deposited at the herbarium of the faculty. The leaves of *M. vulgare* were cut into small pieces and air-dried at room temperature.

### 2.2. Extract Preparation

Two hydroalcoholic extracts of *M. vulgare* were prepared; 100 mg of leaf tissue was ground into powder and then mixed with either 70 mL of ethanol and 30 mL of distilled water to prepare the ethanolic extract (MVE) or with 70 mL of acetone and 30 mL of distilled water for the acetonic extract (MVA). The two extracts were prepared by maceration for 72 h and then filtered and dried in the rotary evaporator. The extracts were stored at 4°C until use.

### 2.3. Total Polyphenol Contents

#### 2.3.1. Total Phenolic Content

The total phenolic content (TPC) was determined using the method described by Slinkard and Singleton [23]. A volume of 100 *µ*L of each extract was mixed with 500 *µ*L of the Folin–Ciocalteu reagent and 400 *µ*L of sodium carbonate solution (75 mg/mL). After incubation at room temperature in the dark for 2 h, the absorbance of the sample was measured at 760 nm using a UV spectrophotometer (Perkin Elmer). TPC was expressed as milligrams of gallic acid equivalents per gram dry weight of the extract (mg GAE/g DW).

#### 2.3.2. Total Flavonoid Content

The total flavonoid content (TFC) was determined according to the method described by Dehpour [24]. A volume of 500 *μ*L of each extract was mixed with 1.5 mL of 95% methanol, 100 *μ*L of 10% AlCl_3_ (m/v), 100 *μ*L of 1 M sodium acetate, and 2.8 mL of distilled water. The mixture was incubated in the dark, at room temperature for 30 min. The blank was prepared by replacing the extract with 95% methanol. The absorbance of the samples was measured at 415 nm using a spectrophotometer (Perkin Elmer). The results are expressed in mg quercetin equivalent per gram dry weight, with reference to a calibration curve prepared using pure quercetin (Sigma-Aldrich (St. Louis, MO, USA).

#### 2.3.3. Total Tannin Content

The total tannin content (TTC) was determined by the method of vanillin as described by Tine [25]. A volume of 0.2 mL of each extract was mixed with 1 mL of vanillin reagent (8% HCl (v/v), methanol at 37% (v/v) and 4% vanillin in methanol (m/v)). The mixtures were first incubated at room temperature, in the dark for 20 min, before measuring the absorbance at 500 nm using a UV spectrophotometer (Perkin Elmer). The results are expressed in mg of ascorbic acid equivalent per gram DW with reference to a calibration curve prepared using pure ascorbic acid (Sigma-Aldrich (St. Louis, MO, USA).

### 2.4. Phytochemical Analysis of the Extract

The phytochemical analysis of *M. vulgare* leaves was carried out by gas chromatography coupled with mass spectrometry (GC-MS) (Model 5973 from Brand Agilent Technologies) according to the protocol described by Birkemeyer [26]; 3 mg of the extract was mixed with 200 *μ*L of N-methyl-N-trimethylsilyl trifluoroacetamide (MSTFA) and incubated for 30 min at 37°C. Helium gas was used as a carrier with a typical pressure range (psi) of 0.9 mL/s. The furnace temperature program was 70°C–270°C at 4°C/min and maintained at 270°C for 20 min. The temperature of the injection was set to 280°C and 290°C and was carried out in a fractionated mode [27].

### 2.5. Evaluation of Antioxidant Activity

#### 2.5.1. DPPH Free Radical Scavenging Activity

The ability of the extracts to scavenge the DPPH free radical was used as a means to estimate their antioxidant capacity, following the protocol of Mssillou [28]. A volume of 100 *µ*L of different concentrations of the extracts (from 1 to 0.007 mg/mL) was added to 750 *µ*L of 0.004% of DPPH. The BHT was used as a positive control. After 30 min of incubation at room temperature and in the dark, the absorbance of the mixture was measured at 517 nm using a spectrophotometer (Jasco V-530). The percentage of inhibition (I%) was calculated using the following equation:

(1) $$\begin{array}{c} I\left(\%\right)=1-\frac{A_{s}}{A_{N}}\times 100, \end{array}$$

where *A*_*N*_ is the absorbance of the negative control (DPPH in ethanol without extract), and *A*_*S*_ is the absorbance of the leaf extract. The IC_50_ is equivalent to 50% of inhibition of DPPH.

#### 2.5.2. Ferric Reducing Antioxidant Power Assay

The antioxidant activity of the different extracts was also evaluated using the ferric reducing antioxidant power method (FRAP) described by Oyaizu [29]. A volume of 0.2 mL of each extract was mixed with 0.5 mL of phosphate buffer (0.2 M, pH 6.6) and 500 *µ*L of potassium ferricyanide [K_3_Fe(CN)_6_] at 1%. The obtained solution was incubated at 50°C for 20 min in a waterbath. The solution was acidified with 0.5 mL of 10% trichloroacetic acid (TCA) and then centrifuged at 3000 rpm for 10 min. A volume of 0.5 mL of the supernatant was mixed with equal volume of distilled water and 100 *µ*L of FeCl_3_ (0.1%), and the absorbance was measured at 700 nm using a spectrophotometer (Jasco V-530). Quercetin was used as a standard. The results were expressed as EC_50_ (mg/mL). The EC_50_ (concentration corresponding to 0.5 of absorbance) was calculated by plotting the absorbance against the corresponding concentration.

#### 2.5.3. Total Antioxidant Capacity

In order to measure the total antioxidant capacity (TAC) of the different extracts, 0.1 mL of each extract was mixed with 1 mL of a reagent solution (0.6 M sulfuric acid + 28 mM sodium phosphate + 4 mM ammonium molybdate). After incubation at 95°C for 90 min, the absorbance at 695 nm was measured using a spectrophotometer (Jasco V-530) using a blank solution containing 0.1 mL of methanol instead of the extract [30]. The total antioxidant capacity was expressed in milligrams of ascorbic acid equivalent per gram of leaf extract (mg AAE/g) using a calibration curve prepared using ascorbic acid.

### 2.6. Antimicrobial Activity

#### 2.6.1. Microbial Strains

The following pathogens were used in the tests: two Gram-positive bacteria (*Bacillus subtilis* and *Staphylococcus aureus*), two Gram-negative bacteria (*Salmonella enterica* and *Escherichia coli*), the human fungal pathogen (*Candida albicans*), and the plant fungal pathogen (*Aspergillus niger*). All pathogens used were obtained from the Hassan II Hospital Center in Fez, Morocco.

#### 2.6.2. Disc Diffusion Method

The antimicrobial activity of the different extracts was tested against the abovementioned pathogens following the method described by Agour [31]. Whatman paper discs of 0.6 cm in diameter were immersed with 10 *µ*L of the extracts (10 mg/disc) and then placed in the surface of Petri dishes containing Mueller-Hinton media already inoculated with 1 × 10^8^ to 2 × 10^8^ CFU/mL of each pathogen. The plates were incubated at 37°C for 24 h for bacterial growth and at 30°C for 48 h for fungal growth. The antimicrobial activity of the extracts was determined by measuring the zone of inhibition around the discs in millimeters. Tetracycline (0.02 mg/disc) and imazalil (0.02 mg/disc) were used as positive controls for bacterial and fungal growth, respectively.

#### 2.6.3. Determination of MICs and MBCs/MFCs

The minimum inhibitory concentration (MIC) and well as the minimum bactericidal/fungicidal concentrations (MBCs/MFCs) were determined based on the microdilution method [32]. A volume of 50 *μ*L of the culture medium was added in each well of the microplate. 100 *μ*L of the extract was added to the first column of wells. Then, microdilutions were carried out by transfer of 50 *μ*L from the wells of the first column to the second one and so on. Inoculation with the pathogens was carried out by depositing 50 *μ*L of the microbial suspension in all the wells. The last column was used as positive control for microbial growth and contained the culture medium and the pathogens, with no extract.

The microplate was incubated at 37°C for 24 h for bacterial growth and at 30°C for 48 h for fungal growth.

Revelation of microbial growth was determined by adding 10 *μ*L of resazurin (5 mg/mL) to the wells and incubating the plate at 37°C for 30 min. Color change of the wells indicates microbial activity. The MBC/MFC was determined by spot inoculation (2–4 *μ*L) on nonselective agar (MHA divided into numbered squares) from the wells which were found negative.

### 2.7. Statistical Analysis

All tests were conducted in triplicates, and values were expressed as mean ± standard deviation. The statistical analysis of the results was performed using GraphPad Prism software (version 5), by one-way analysis of variance (ANOVA), followed by the Tuckey test, and differences at *P* < 0.05 were considered significant.

## 3. Results and Discussion

### 3.1. Yield of Extracts

A yield of 13% was obtained for both extracts regardless of the type of solvent used. Previous studies using *M. vulgare* have reported similar results using other solvents and/or extraction methods [21, 33].

### 3.2. Phytochemical Content

**Figure 1:**
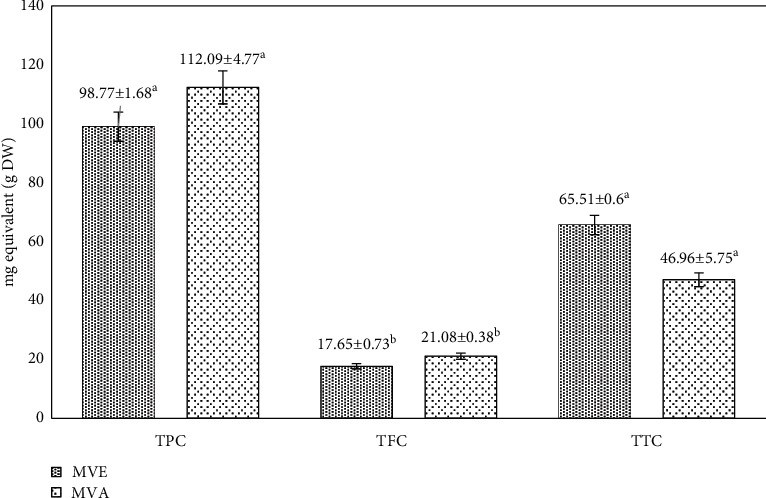
TPC, TFC, and TTC in mg (gallic acid, quercetin, and ascorbic acid, respectively) equivalent/gram dry weight of two extracts from *Marrubium vulgare* L. The results are expressed by means ± standard deviation, and values with different letters are significantly different (*P* < 0.05).

The total phenolic, flavonoid, and tannin contents in the two extracts were expressed in mg equivalent/gram of the plant DW (Figure 1). The calibration curves of gallic acid (*y* = 0.7333*x* + 0.028; *R*^2^ = 0.9938), quercetin (*y* = 0.857x−0.011; *R*^2^ = 0.970), and ascorbic acid (*y* = 0.275*x* + 0.0185; *R*^2^ = 0.9908) were performed at 1 mg/mL. The MVA extract contained high level of TPC and TFC (112.09 ± 4.77 and 21.08 ± 0.38 mg Eq/g DW, respectively), compared to MVE (98.77 ± 1.68 and 17.65 ± 0.73 mg Eq/g DW, respectively). However, MVE was rich in tannins (65.51 ± 0.6 mg Eq/g DW) compared to MVA (46.96 ± 5,75 mg Eq/g DW). The TPC, TFC, and TTC of *M. vulgare* leaves from a neighboring country, Algeria, have been measured and showed a difference in the content [34, 35]. These differences can be explained by the differences in genotype, environment, plant organ, and type of solvent used in the study [13]. In particular, the influences of climatic, edaphic, and genetic factors on the biosynthesis and accumulation of TPC and TFC have been well established [9, 10].

### 3.3. GC/MS Analysis

The chemical composition of the extract of *M. vulgare* was determined by GC/MS and revealed the presence of 13 compounds with a total of 96.14% (Table 1). The major compound was the malic acid (tms) with a percentage of 22.57% followed by silanol, trimethyl-, phosphate (3 : 1) (19.08%), and other compounds such as galactose oxime hexaTMS with a lowest percentage achieved 9.94%. The presence of malic acid (tms) in *M. vulgare* has been reported previously [36], and its antioxidant and antimicrobial activities are documented [37–39]. The phytochemical composition of the plant extract influences its bioactive power, and there is a correlation between the chemical composition of the extract and its biological activities [40, 41].

It should be mentioned that some compounds identified in *M. vulgare* have been identified in other plants such as cyclotetrasiloxane and octamethyl, identified in the methanol extract of *Dillenia scabrella* Roxb. (Dilleniaceae) and the methanolic extract of *Pulicaria undulata* L. (Asteraceae) [42, 43]. Silanol, trimethyl-, phosphate was identified in *Anacardium occidentale* nut [44] also in the extract of *Terminalia bellirica* (Combretaceae) seed [45]. Spiro[1,3-dithiane-2,4′-cyclopent[c]isoxazolidine] was detected in the ethanol extract from the aerial parts of *Eryngium carlinae* F. Delaroche (Apiaceae) [46].

### 3.4. Antioxidant Activity

#### 3.4.1. DPPH Free Radical Scavenging Activity

Both extracts exhibited significant DPPH scavenging activity, but the MVE showed significant free radical activity compared to the MVA extract. The MVE extract (IC_50_ = 52.04 ± 0.2 *µ*g/mL) has a better reducing power of free radicals compared to the MVA extract (IC_50_ = 60.57 ± 0.6 *µ*g/mL) (Table 2), but much lower than the positive control BHT (IC_50_ = 9.28 ± 0.2 *µ*g/mL). This is consistent with other previously reported studies [47, 48].

The reduction of free radicals in a solution is performed by the exchange of electron and hydrogen atoms, and the difference between the two extracts can be explained by the difference in the composition of polyphenols caused by the extraction solvents [49].

#### 3.4.2. Ferric Reducing Power

This method consists of the reduction of Fe^3+^ ions to Fe^2+^ in the presence of an antioxidant agent by the intermediate of donating hydrogen atom [50]. The results of the evaluation of ferric reducing power of the extracts of *M. vulgare* are given in Table 2. The MVE extract exhibited a better antioxidant activity (EC_50_ = 4.51 ± 0.5 mg/mL) compared to MVA (EC_50_ = 6.43 ± 0.0411 mg/mL). The activity of both extracts was lower than the activity of the positive control ascorbic acid (EC50 = 2.19 ± 0.9 mg/mL). Previous studies have reported the antioxidant potential of *M. vulgare* [34, 51]. The difference in the reducing power of iron from one extract to another may be due to the difference in the chemical composition and the content of phenolic compounds [52].

#### 3.4.3. Total Antioxidant Capacity

The total antioxidant activity of the two extracts of *M. vulgare* was evaluated by the method of phosphomolybdenum [30]. This method evaluates the capacity of the extract to convert Mo (VI) to Mo (V) [30]. The results of this test are expressed in Table 2. The results indicate that the hydroacetonic extract has stronger total antioxidant capacity (273.52 ± 16.67 mg AAE/g) compared to the hydroethanolic extract (218.58 ± 19.24 mg AAE/g). This antioxidant may be due to its chemical composition and its richness in antioxidants.

### 3.5. Antimicrobial Activity

#### 3.5.1. Disc Diffusion Method

The disc diffusion method was employed in order to determine the extent of inhibition of the extracts. The MVE extract showed an activity against all the strains tested with an inhibition zone ranging from 7.33 ± 0.33 mm to 11.66 ± 0.66 mm. Both MVA and MVE extracts showed a comparable effect against each other. The MVE extract appeared to exhibit a better activity against *S. enterica* than *E. coli*. Moreover, the MVA extract has more effective activity against *C. albicans* than the MVE extract (Table 3). The antimicrobial potential of *M. vulgare* against several other pathogens has been documented previously [53–55].

#### 3.5.2. MIC and MBC/MFC of Extracts

The MVE exhibited a strong inhibitory effect against *S. aureus* (MIC of 1.87 mg/mL), followed by *B. subtilis* and *E. coli* (MIC of 2.5 mg/mL) (Table 4). A very low effect of the MVE extract was observed against *S. enterica* (MIC of 5 mg/mL) and *A. niger* (MIC of 10 mg/mL). On the contrary, the MVA extract showed a better effect against *C. albicans* (MIC of 0.93 mg/mL and MBC of 1.75 mg/mL) compared to the MVE extract. The MVA extract also showed better activity against *S. enterica* (MIC and MBC of 2.5 mg/mL) compared to the MVE extract. The activity of the MVA extract against *B. subtilis* and *E. coli* was low compared to the MVA extract. The MVA extract had a very weak effect against *S. aureus* (MIC/MBC of 10 mg/mL) when compared to the MVE extract (MIC/MBC of 1.87 mg/mL). Also, both extracts had no effect against *A. niger* (Table 4).

Previous studies have reported that the extracts of *M. vulgare* are not effective against Gram-negative bacteria [56, 57]. The differences in the ability of this plant to react against pathogenic strains can be explained by the locality from where this plant was harvested and the environmental effects on its chemical composition. In addition, the method of extraction the solvent used can have an effect on the antimicrobial activity of the plant extract.

## 4. Conclusion

*Marrubium vulgare* L. MVE and MVA leaf extracts are rich in important chemical compounds especially malic acid. The leaves also contain high level of phenolic compounds, which confer to the plant as an important antioxidant power and antimicrobial activity against nosocomial strains.

## Figures and Tables

**Table 1:** Phytochemical composition of *M. vulgare* extract.

Peak	Compounds	Formula	RT (min)	% area
1	Cyclotetrasiloxane, octamethyl- (CAS)	C_8_H_24_O_4_Si_4_	8.22	3.02
2	Silanol, trimethyl-, phosphate (3 : 1)	C_9_H_27_O_4_PSi_3_	9.16	19.08
3	4-p-Tolylcyclohexene	C_13_H_16_	10.45	3.77
4	Malic acid (tms)	C_13_H_30_O_5_Si_3_	10.80	22.57
5	Xylitol 5TMS	C_20_H_52_O_5_Si_5_	12.42	3.00
6	Spiro[1,3-dithiane-2,4′-cyclopent[c]isoxazolidine]	C_9_H_15_NOS_2_	12.99	4.63
7	D-Xylofuranose, 1,2,3,5-tetrakis-O-(trimethylsilyl)-	C_17_H_42_O_5_Si_4_	13.05	2.97
8	D-Galactose 5TMS	C_21_H_52_O_6_Si_5_	13.47	4.35
9	Galactose oxime hexaTMS	C_24_H_61_NO_6_Si_6_	13.73	9.94
10	Alpha-D-galactopyranose, 1,2,3,4,6-pentakis-O-(trimethylsilyl)-	C_21_H_52_O_6_Si_5_	13.85	9.10
11	6,7-Bis(trimethylsilyl)-1,3-dimethoxyisoquinoline	C_17_H_27_NO_2_Si_2_	14.25	2.90
12	Trimethylsilyl-meso-inositol	C_24_H_60_O_6_Si_6_	14.63	7.00
13	Beta-D-galactofuranoside, ethyl 2,3,5,6-tetrakis-O-(trimethylsilyl)-	C_20_H_48_O_6_Si_4_	18.17	3.81

Total				96.14

RT, retention time.

**Table 2:** Antioxidant activities of *M. vulgare* extracts by DPPH, FRAP, and TAC methods.

	DPPH IC_50_ (*µ*g/mL)	FRAP EC_50_ (mg/mL)	TAC (mg AAE/g DW)
MVE	52.04 ± 0.2^a^	4.51 ± 0.5^a^	218.58 ± 19.24^b^
MVA	60.57 ± 0.6^a^	6.43 ± 0.0411^a^	273.52 ± 16.67^b^
Ascorbic acid	—	2.19 ± 0.9^a^	—
BHT	9.28 ± 0.2^b^	—	—

Values in the same column sharing different letters are significantly different at *P* < 0.05.

**Table 3:** Inhibition zone of MVE and MVA extracts of *M. vulgare* leaves against pathogenic microbial strains.

Microbial strains	Inhibition zone diameter (mm)			
	MVE	MVA	Tetracycline	Imazalil
Gram-positive				
*Bacillus subtilis*	10.33 ± 0.33	10 ± 0.5	30 ± 1.5	NT

Gram-negative				
*Escherichia coli*	11.66 ± 0.66	NA	8.33 ± 0.66	NT
*Salmonella enterica*	8 ± 0.5	NA	19 ± 0.5	NT

Fungus				
*Candida albicans*	8 ± 0.5	6 ± 0.5	NT	14 ± 1
*Aspergillus niger*	7.33 ± 0.33	NA	NT	14.33 ± 0.66

^*∗*^Results are expressed by means ± standard deviation; for each value, the test was performed in triplicate (*n* = 3). NT, not tested; NA, not active.

**Table 4:** MICs and MBC/MFCs (mg/mL) of MVE and MVA extracts.

Pathogens	MIC^a^ and MBC/MFC^b^ (mg/mL)							
	MVE		MVA		Tetracycline		Imazalil	
	MIC	MBC	MIC	MBC	MIC	MBC	MIC	MFC
Gram-positive bacteria								
*Bacillus subtilis*	2.5	2.5	3.75	3.75	0.25	0.25	NT	NT
*Staphylococcus aureus*	1.87	1.87	10	10	0.062	0.25	NT	NT

Gram-negative bacteria								
*Escherichia coli*	2.5	2.5	3.75	3.75	0.25	0.5	NT	NT
*Salmonella enterica*	5	5	2.5	2.5	0.5	0.75	NT	NT

Fungal species								
*Candida albicans*	1.75	2.5	0.93	1.75	NT	NT	0.05	0.05
*Aspergillus niger*	10	>10	10	>10	NT	NT	0.1	0.1

^a^Minimum inhibitory concentration. ^b^Minimum bactericidal/fungicidal concentration. NT, not tested.

## Data Availability

The data used to support the findings of this study are available from the corresponding author upon request.
